# Two-minute walk test: Reference equations for healthy adults in China

**DOI:** 10.1371/journal.pone.0201988

**Published:** 2018-08-09

**Authors:** Jia Zhang, Xiaoshu Chen, Shiwei Huang, Yi Wang, Wei Lin, Rui Zhou, He Zou

**Affiliations:** 1 Department of Inspection Medical, Wenzhou People’s Hospital, the Wenzhou Third Clinical Institute Affiliated with Wenzhou Medical University, Wenzhou, Zhe Jiang, China; 2 Department of Cardiovascular Medicine, Wenzhou People’s Hospital, the Wenzhou Third Clinical Institute Affiliated with Wenzhou Medical University, Wenzhou, Zhe Jiang, China; The Ohio State University, UNITED STATES

## Abstract

**Background and objective:**

Although the six-minute walk test (6MWT) is widely used as a measure of exercise capacity, it may not be applicable in some settings and populations. This issue has led to increased use of the two-minute walk test (2MWT) to assess exercise capacity. The main objective of this study is to establish reference equations for the two-minute walk distance (2MWD) in healthy Chinese adults aged 18–85 years.

**Methods:**

A total of 973 volunteers took part in the study. We obtained verbal consent from all participants before the test, and the study design was approved by the ethics committees of Wenzhou People's Hospital. The participants performed two 2MWTs using a standardized protocol, and the longer distance was used for further analysis. Stepwise multiple regression analysis was performed using age, height and weight as independent variables and was used to establish the reference equations for the 2MWD in the male and female groups.

**Results:**

The mean walking distance for all participants was 199.1±25.81 m. Age and height were identified as independent factors that influenced the 2MWD, and they explained 35% and 34% of the variance in distance for the male and female groups, respectively.

**Conclusion:**

This study resulted in determination of reference equations for predicting the 2MWD in healthy Chinese adults. These 2MWD standards will provide useful references for medical care in some settings and populations.

## Introduction

Walking tests are easy, safe, inexpensive tools for measuring functional exercise capacity in clinical practice and research. The American Thoracic Society (ATS) specifically recommended the 6-minute walk test (6MWT) and published guidelines for its administration in 2002.[1] Although the 6MWT is widely used in patients with stable cardiorespiratory disease,[2] it may not be applicable in patients who are hospitalized with an acute exacerbation of chronic obstructive pulmonary disease.[3] The two-minute walk test (2MWT) is highly related to the 6MWT.[4–6] Therefore, in some populations, especially those with end-stage cardiorespiratory disease or those who are hospitalized for an acute exacerbation of chronic obstructive pulmonary disease, the 2MWT may be an appropriate alternative to the 6MWT for assessing functional exercise capacity. This issue has led to increased use of the 2MWT to assess exercise capacity. At present, the 2MWT has been used in several different diseases and populations (e.g., the frail elderly,[6, 7] recent cardiac surgery,[8] obesity,[9] stable COPD,[10, 11] recent hip fracture,[12] circulatory insufficiency,[8] lower limb amputations,[13] and multiple sclerosis[14]). Prediction equations for the two-minute walk distance (2MWD) derived from samples of different ethnicities[15–17] have been established, but these equations differ considerably. Although participants in the study by Mirza et al[17] included Chinese people, the sample size of Chinese participants was relatively small (N = 23) and insufficient to identify potential interactions between the 2MWD and other variables. Moreover, the model in their study cannot be sufficiently validated. Additionally, participants included in studies by Selman et al [15]and Mirza et al [17] were not all healthy people. Thus, for these reasons, the previous published equations may be not suitable for Chinese people.

The aims of this study were as follows: (1) to establish reference equations for the 2MWD in healthy Chinese adults aged 18–85 years, (2) to test its reproducibility, and (3) to compare the measured 2MWD of our cohort with the predicted 2MWD based on previously published equations derived from foreign studies.[15–17]

## Methods

### Study participants

A total of 973 volunteers took part in the study. This study was performed after we obtained ethical approval from the ethics committee of Wenzhou People’s Hospital. The study proposal was provided to the reviewers to assure them that there were no ethical issues prior to the ethical approval. The ethics committee approved the verbal consent by considering that the study does not cause serious harm to the participants. The researchers needed to explain the purpose of the study to each participant and obtain verbal informed consent before the study. A convenience sample of volunteers was tested, and we collected data over a 47-month period from December 2013 to October 2017 in this cross-sectional study. The volunteers included students and teachers at two local universities, employees of two local private companies, community residents and workers and medical personnel from a public hospital. Inclusion criteria were as follows: 18–85 years of age, a body mass index (BMI) ≤30 kg/m^2^, a body mass index (BMI) ≥ 18 kg/m^2^_,_ no organic disease and no problems that affected walking ability.

### Physical examination

Before the test, the researcher recorded participants’ resting demographic information and anthropometric data, such as age, height, weight and BMI (weight/height^2^). As previously described in detail,[18] the researchers needed to know the type, frequency, and duration of participants’ exercise activity for the month before the study. If participants had performed more than 20 minutes of lower limb exercises, 3 times per week, in the last month, they were classified as “physically active”.[19] Participants who did not meet these criteria were classified as “sedentary”. Lung function evaluations, such as forced vital capacity (FVC), forced expiratory volume in one second (FEV_1_) and FEV_1_/FVC, were assessed with a standard portable spirometer according to the ATS guidelines.[20]

### Two-minute walk test

The 2MWT is a modified version of the ATS guidelines for the 6MWT.[1] The 2MWT was performed in an enclosed, wide, long, flat, 30-m corridor. Prior to the test, participants were instructed to rest in a chair near the starting line for at least 10 minutes while some data (i.e., heart rate, blood pressure and oxygen saturation) were measured and recorded by the researcher. Blood pressure was calculated according to National Institutes of Health (NIH) guidelines.[21] Then, participants were asked to walk back and forth on the course as far as possible without running for a 2-minute period. In the case of severe symptoms of exercise intolerance, participants were allowed to slow down or rest, but once they recovered, we encouraged them to continue walking again as soon as possible. The investigator informed participants of the time left until the end of the 2MWT and gave them standardized encouragement (i.e., “good job; there is 1 minute left” and “good job; there are 30 seconds left”). The distance covered over the two minutes was recorded as the 2MWD. Two 2MWTs were performed at a two-hour interval and administered by the same investigator, and the longer 2MWD was used for further analysis.

## Statistical analysis

Descriptive analysis of demographic features was conducted. The main variables in the study were normally distributed and assessed by the Kolmogorov-Smirnov test. The results are presented as the mean ± standard deviations or range. The characteristics of categorical variables are shown as numbers and percentages. Independent Student’s *t* test was assessed to determine the associations between the 2MWD and categorical variables (e.g., activity and gender). The measured 2MWD of our cohort was compared with the predicted 2MWD based on previously published equations derived from foreign studies[15–17] using paired *t*-tests. The repeatability of the two 2MWTs was examined using the intra-class correlation coefficient (ICC) and Bland Altman analysis[22]. We first used univariate analysis with Spearman’s correlation test to evaluate the correlation between the 2MWD and categorical variables (i.e., age, height, weight and BMI), and then used forward stepwise multiple regression analysis to establish the reference equations for the 2MWD. The most significant variable was added to the model at each step, and the process continued until no additional further statistically significant variables could be added. A p-value>0.05 was used to determine whether a variable was entered and removed. Variance inflation factors were used for multicollinearity analysis. Statistical Package for the Social Sciences (SPSS 17.0) was used for all analyses. The threshold of statistical significance for all analyses was set at p<0.05.

## Results

### Demographic information, anthropometric data and lung function

A total of 973 volunteers took part in the study, one hundred and forty-two were excluded (due to physical discomfort, undiagnosed hypertension, abnormal pulmonary function, BMI>30 kg/m^2^ or <18 kg/m^2^, a resting heart rate<50 bpm or > 100 bpm and physician-diagnosed organic disease). Ultimately, 831 participants (410 males and 421 females) completed the 2MWT, and no participants prematurely terminated the test or required a rest during the test. The baseline characteristics and 2MWT results of study participants are summarized in Table 1. We found significant gender differences in height, weight and BMI. The male group was significantly taller and heavier than the female group.

**Table 1:** Characteristics and 2MWT results of study subjects.

Characteristic	Males (n = 410)	Females (n = 421)	Total (n = 831)
Age, years	45.2±15.82 (18–85)	46.4±15.93 (18–85)	45.8±15.88 (18–85)
Height, cm	168.9±6.47 (150–188)	158.4±5.00 (145–172)	163.6±7.81 (145–188)
Weight, kg	66.0±8.50 (45–96)	56.7±7.61 (43–80)	61.3±9.30 (43–96)
BMI, kg/m^2^	23.2±2.78 (18–30)	22.6±2.87 (18–30)	22.9±2.84 (18–30)
Sedentary, m	208.6±23.49 (137–259)	182.60±23.55 (116–236)	194.9±26.84 (116–259)
Physically active, m	214.3±22.55 (143–266)	190.2±20.00 (139–243)	202.5±24.47 (139–266)
2MWD, m	211.9±23.1(137–266)	186.7±22.01 (116–243)	199.1±25.81 (116–266)

Values are expressed as the mean±SD (range); sedentary: the 2MWD in the sedentary group; physically active: the 2MWD in the physically active group

### Two-minute walk distance

The mean 2MWD for all participants was 199.1±25.81 m. Age and gender stratified values of the 2MWD are summarized in Table 2. The mean distance was 211.9±23.1 m for males and 186.7±22.01 m for females, and the difference was significant (p<0.001). The mean distance was 194.9±26.84 m for sedentary participants and 202.5±24.47 m for physically active participants. Additionally, the physically active participants walked significantly longer distances than did sedentary participants (p<0.001). We also found a significant gender difference in terms of blood pressure, heart rate and oxygen saturation. The mean distances walked during the first test session and the second test session were 192.4±25.32 m and 196.5±25.86 m, respectively. The reliability of the 2MWT was good (ICC = 0.88).

**Table 2:** Age and gender stratified values of the 2MWD.

Age, years (n)	Males (n = 410)	Females (n = 421)	p-value*	Total (n = 831)
18–29 (167)	225.5 (188–266)	199.1 (142–233)	<0.001	212.4 (142–266)
30–39 (132)	221.6 (177–258)	198.5 (161–243)	<0.001	212.1 (161–258)
40–49 (154)	217.9 (172–253)	192.7 (157–230)	<0.001	202.7 (157–253)
50–59 (202)	208.7 (170–248)	184.1 (152–226)	<0.001	196.9 (152–248)
60–69 (129)	196.1 (162–239)	172.4 (139–223)	<0.001	183.6 (139–239)
70–85 (47)	165.5 (137–185)	148.8 (116–183)	<0.001	156.3 (116–185)

Values are expressed as the mean (range)

*p-value between males and females

2MWD: two-minute walk distance.

### Associations with the two-minute walk distance

According to univariate linear regression analysis, the variables (i.e., age, height and BMI) showed a significant relationship with the 2MWD. The relationships between the 2MWD and age, height and BMI in the male and female groups are summarized in Table 3. The variables (age, height and BMI) were used in stepwise multiple regression analysis. Age and height were identified as independent factors that influenced the 2MWD and explained 35% and 34% of the variance in distance for the male and female groups, respectively (Table 4).

**Table 3:** Pearson correlations between the variables and 2MWD.

Variable	Males (n = 410)		Females (n = 421)	
	r value	p-value	r value	p-value
Age, years	-0.571	<0.001	-0.566	<0.001
Height, cm	0.421	<0.001	0.367	<0.001
Weight, kg	0.058	NS	0.013	NS
BMI, kg/m^2^	-0.215	<0.001	-0.157	0.001

2MWD: two-minute walk distance; r value: Pearson’s correlation coefficient; BMI: body mass index.

**Table 4:** Results of stepwise multiple linear regression analysis of independent variables that explained the 2MWD.

	Males			Females		
	B	SE	p-value	B	SE	p-value
Constant	123.252	28.549	<0.001	108.278	31.693	0.001
Age, years	-0.699	0.065	<0.001	-0.691	0.060	<0.001
Height, cm	0.711	0.160	<0.001	0.698	0.192	<0.001
R	0.598			0.584		
Adjusted R^2^	0.354			0.338		

B, unstandardized coefficients.

The reference equations for the 2MWD were as follows:

$$\begin{array}{l} Male\;:\;2MWD\left(m\right)=123.252-\left[age\left(yr\right)\times 0.699\right]+\left[height\left(cm\right)\times 0.711\right];\;r^{2}=0.354\\ Female\;:\;2MWD\left(m\right)=108.278-\left[age\left(yr\right)\times 0.691\right]+\left[height\left(cm\right)\times 0.698\right];\;r^{2}=0.338 \end{array}$$

### Comparison with published reference equations

The 2MWDs measured in our study were compared with the predicted 2MWD derived from three published equations. The reference equations from Selman et al[15] and Mirza et al[17] overestimated the walking distances of our participants and the reference equations by Bohannon et al[16] underestimated the distances. The differences between the measured 2MWD and the predicted 2MWD for the same age range based on the equations reported in the studies by Selman et al[15], Bohannon et al[16] and Mirza et al[17] were -9.9±19.48 m, 6.0±19.65 m and -15.3±18.17, respectively.

## Discussion

**Fig 1:**
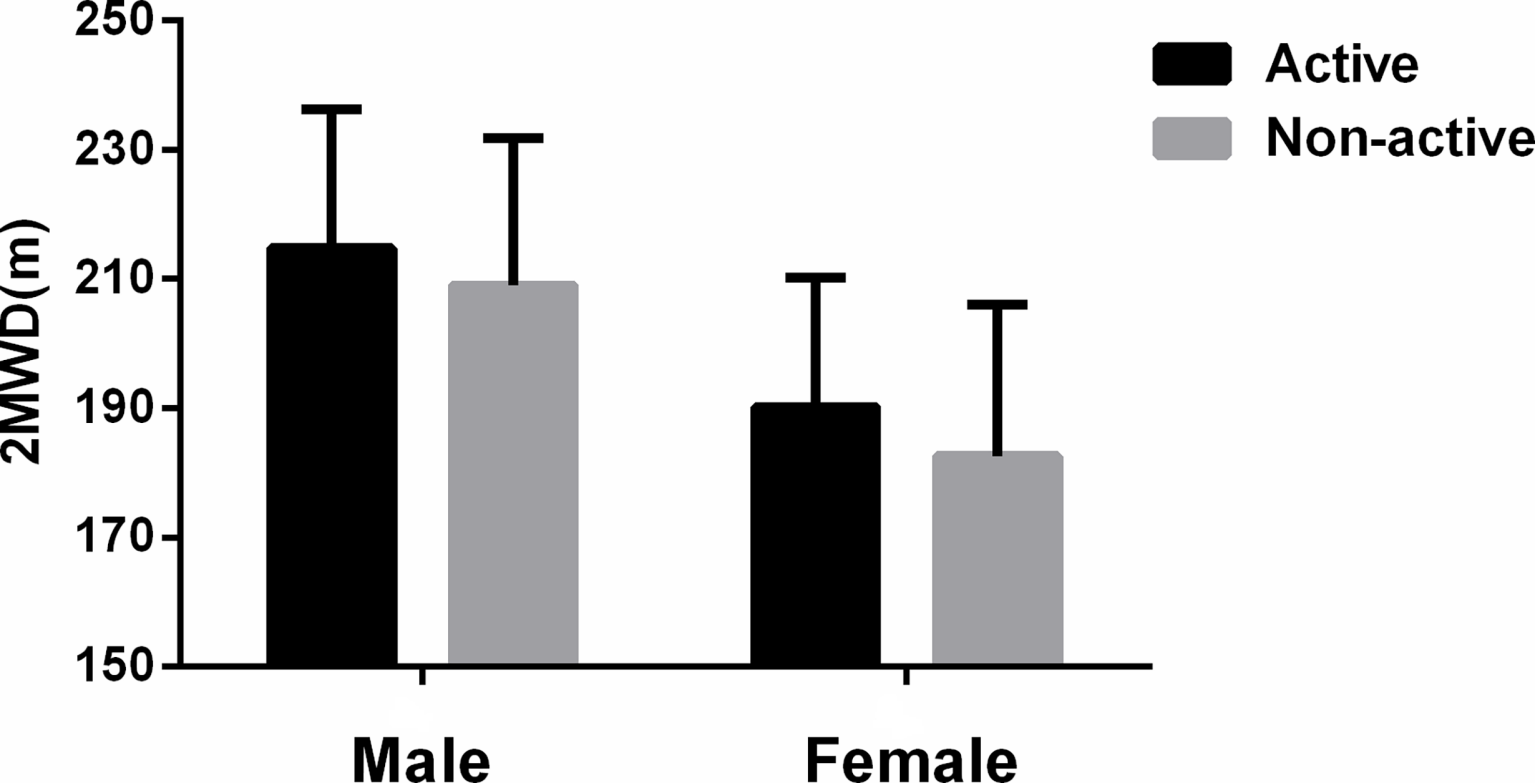
The effect of activity on the 2MWD in males and females.

To the best of our knowledge, this study is the first to describe the 2MWD in healthy Chinese adults aged 18–85 years. The mean 2MWD was 199.1±25.81 m, and the male group walked a longer distance than did the female group, possibly because the males were taller and had higher levels of physical activity and greater muscle mass. Compared with sedentary participants, physically active participants walked significantly longer distances. In our study, there were significant differences in the 2MWD between the active group and the sedentary group (Fig 1). A significant direct relationship has been found between physical exercise and muscle strength in exercise physiology studies.[23] Conversely, a sedentary lifestyle usually influences muscle mass and muscle metabolism and leads to a decline in physical energy[23], which could explain why the mean 2MWD of sedentary participants in our study was significantly shorter than that of physically active participants.

**Fig 2:**
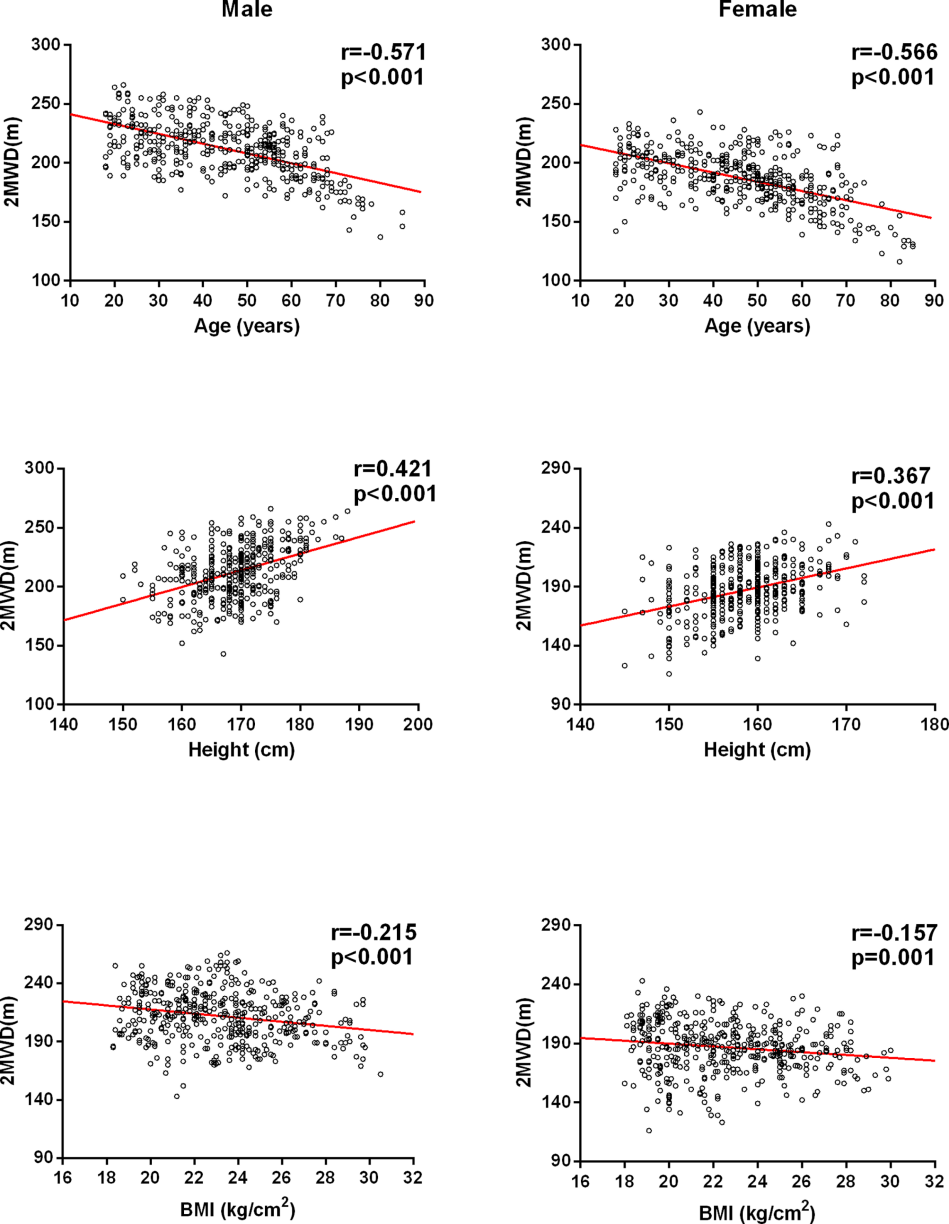
The relationship between the 2MWD and independent variables in males and females.

There was some relationship between independent variables (age, height and BMI) and the 2MWD for males and females (Fig 2). There was a significant inverse relationship between the 2MWD and age, and age was the predominant variable in the regression equation for our participants. This association could be due to the gradual decrease in muscle mass, muscle strength and maximal oxygen uptake with age. Height was strongly correlated with the distance walked and was the predominant variable in the regression equation for our participants. This result was likely observed because taller height is associated with a longer stride, which makes walking more efficient. We found that BMI was significantly correlated with the 2MWD in males and females. However, BMI was not represented in the final regression equation, possibly because overweight and underweight participants were excluded from our study. In this study, categorical variables (age, height, weight and BMI) were used for stepwise multiple regression analysis. Age and height were identified as independent factors that influenced the 2MWD, and they explained 35% and 34% of the variance in distance for the male and female groups, respectively.

**Fig 3:**
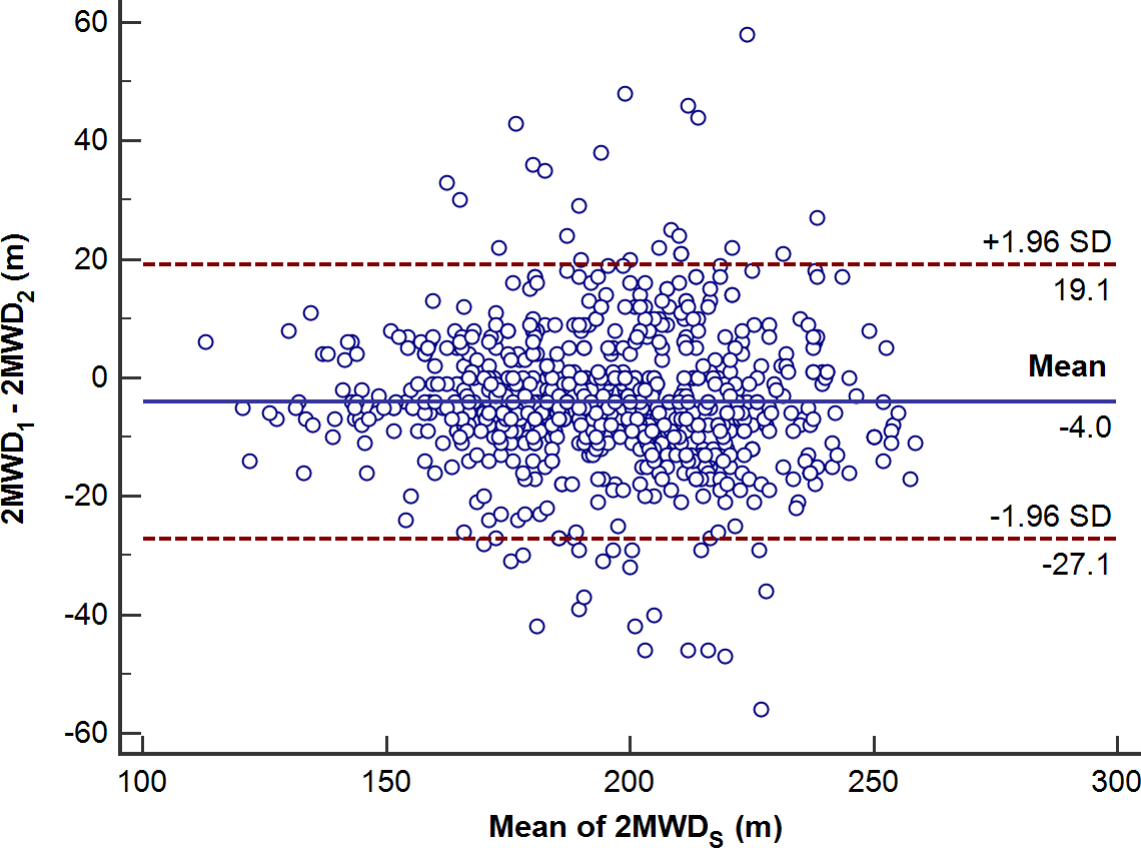
Bland–Altman plot showed the mean difference between the first and second performance on the 2MWT.

By measuring the 2MWT performance on 2 occasions, we found that the mean distances walked during the second test session were longer than those walked during the first test session. The finding is in agreement with previous findings for the 6MWT.[24–26] The increase in distance may be due to overcoming anxiety, improved coordination and finding an optimal stride length. Though the distance walked during the second performance of the 2MWT in our study was increased compared with that during the first performance, the reliability of the 2MWT was good (ICC = 0.88). Previous studies have demonstrated the reliability of the 2MWT[11, 16, 27], which is consistent with the findings of our study. The Bland–Altman plot showed the mean difference between the first and second performance on the 2MWT (Fig 3). Forty-four participants had error values outside the 95% confidence interval (CI), and twenty-three participants presented an increase in the second 2MWT, which might have been caused by familiarization with the test. Twenty-one participants presented a decreased distance in the second 2MWT, which might be due to high performance on the first 2MWT that led to greater fatigue during the second test.

We observed that the reference equations for the 2MWD based on other populations were not suitable for Chinese people. The differences are commonly caused by differences in anthropometric factors, demographic characteristics, participants’ racial backgrounds and the test protocol. The reference equations by Bohannon et al[16] underestimated the walking distances of our participants. Compared with participant in our study, participants in the study by Bohannon et al[16] were taller and heavier, and there were some differences in the test protocol. For instance, encouragement was provided during the test every 60 seconds, the corridor was 15.2 m and only a subset of participants was tested twice in the study of Bohannon et al.[16] However, in our study, encouragement was provided during the test every 30 seconds, the corridor was 30 m, the 2MWT was performed twice, and the longer 2MWD was used for further analysis. The inclusion of the frequency of encouragement during the test[28], the length of the corridor[29] and a practice test[30] have each been demonstrated to influence the 6MWD, and these factors also likely influence the 2MWD. The reference equations by Selman et al[15] and Mirza et al[17] overestimated the walking distances of our participants. Although compared with our study, the studies by Selman et al[15] and Mirza et al[17] have a similar test protocol (i.e., the frequency of encouragement during the test, the length of the corridor and a practice test), participants in the studies by Selman et al[15] and Mirza et al[17] were taller and heavier than those in our study. In addition to participants’ levels of daily physical activity, their attitudes and psychological factors may influence the distance walked.

Our study has some limitations. First, although the current study had a relatively large sample size, the sample was one of convenience and relatively few participants were over seventy years old. Second, the retest was performed within two hours of the test. Third, we did not measure blood pressure and heart rate immediately after participants completed the 2MWT. Thus, future prospective studies are needed to solve these problems.

In conclusion, this study resulted in determination of reference equations for predicting the 2MWD in healthy Chinese adults. These 2MWD standards will provide useful references for medical care in some settings and populations.

## Supporting information

S1 Dataset Subjects’ characteristics and 2MWT outcomes. (XLSX)

## Abbreviations

6MWT: six-minute walk test
6MWD: six-minute walk distance
2MWT: two-minute walk test
2MWD: two-minute walk distance
BMI: body mass index
FEV_1_: forced expiratory volume in the first second
FVC: forced vital capacity
HR: resting heart rate
SpO_2_: resting oxygen saturation
SBP: resting systolic blood pressure
DBP: resting diastolic blood pressure
ICC: intra-class correlation
SD: standard deviation
SPSS: Statistical Package for the Social Sciences
